# Effect of Bleaching Agents on Healthy Enamel, White Spots, and Carious Lesions: A Systematic Review and Meta-Analysis

**DOI:** 10.3390/dj12050140

**Published:** 2024-05-11

**Authors:** Grigoria Gkavela, Vlassios Kakouris, Eftychia Pappa, Christos Rahiotis

**Affiliations:** Department of Operative Dentistry, Dental School, National and Kapodistrian University of Athens, 11527 Athens, Greece; grigoriagk@dent.uoa.gr (G.G.); vlassiosk@dent.uoa.gr (V.K.); effiepappa@dent.uoa.gr (E.P.)

**Keywords:** systematic review, bleaching agents, carious lesions, white spots, enamel microstructure

## Abstract

This systematic review examines studies focusing on tooth bleaching and its effects on healthy enamel or incipient caries and bacterial adhesion. The aim is to explore the impact of different bleaching agents on incipient caries lesions and healthy enamel. Clinical studies, in vitro studies, and observational studies that compared at least two groups were included. A search strategy was used to select studies from the MEDLINE via Pubmed and Scopus databases. Two evaluators performed data extraction, screening, and quality assessment independently. Only studies written in English were included. From 968 initial records, 28 studies were selected for a full-text evaluation. Of these, 7 studies were classified as cluster 1 (bacterial adherence on teeth), 12 studies as cluster 2 (no bacteria involved), 4 studies as cluster 3 (no teeth deployment), and 5 clinical studies were cluster 4. Of the selected studies, 6 (21.4%) supported increased bacterial attachment capacity and cariogenic dynamics, 4 (14.3%) decreased adhesion and cariogenic activity, 7 (25%) showed no difference, and 11 (39.3%) followed a different methodological approach and could not be categorized. The risk of bias appeared to be high, mainly because of the different methodologies in the studies, so we cannot reach a confident conclusion. Nevertheless, as far as carbamide peroxide bleaching is concerned, there does not seem to be a clinically significant alteration, neither in microorganism counts nor in enamel microstructure.

## 1. Introduction

Nowadays, tooth bleaching is a popular, non-invasive aesthetic treatment, as many people seek a healthier and more youthful appearance. More adults are undergoing tooth bleaching to improve the esthetic appearance of their teeth [1]. In a study from 2013, approximately 66% of the participants were unsatisfied with their tooth color [2]. 

Many in vitro and clinical studies have been performed over the years to assess clinical issues such as longevity of the outcome, patient satisfaction, and sensitivity [3,4,5,6,7,8,9,10,11]. 

Among the various vital bleaching techniques currently available to clinicians, home bleaching is considered in the literature to be the most effective treatment and ensures predictable color stability [3,9,11]. Many studies conclude that in-office bleaching is less effective than home bleaching [5,8]. However, many patients and dentists still prefer in-office bleaching [12,13] due to the reduced application time. Also, the efficacy of various concentrations of bleaching agents has been researched extensively. Recent meta-analyses suggest that 10% carbamide peroxide is the most efficient bleaching agent, with negligible adverse effects and sensitivity [14,15]. 

As dental bleaching is a common practice, it is essential to study the potential effects of this treatment on enamel, bacterial adhesion, and incipient caries. The effect of dental bleaching on enamel has been investigated in numerous studies. For example, due to home bleaching with 10% carbamide peroxide, enamel microhardness and structure alterations may affect tooth resistance to mechanical forces [16]. Still, there are other properties that we must take into consideration, such as enamel microroughness [16].

White spot lesions are one of the most frequent adverse effects of orthodontic treatment; nevertheless, the efficacy of various interventions for masking these lesions is yet to be addressed and researched. Bleaching agents have an efficient masking effect on white spots as well as incipient caries lesions [17,18].

An incipient caries causes demineralization underneath a superficially intact layer. The scattering of light is different than for the surrounding healthy enamel, giving the spot a chalky white appearance [19]. However, applying carbamide or hydrogen peroxide onto a demineralized exterior layer raises many concerns since previous studies have shown that they affect the contents of minerals in enamel [20,21,22]. However, the long-term impact of bleaching methods on such carious lesions remains uncertain.

Tooth bleaching is associated with a statistically significant short-term decrease in biofilm accumulation and improvement in plaque index [23]. It is still unclear if this decrease is attributed to the effect of bleaching agents or whether the people who have their teeth whitened are at the time more aware of their oral hygiene. However, no data are available to evaluate the long-term effects [23] of this treatment on oral health.

The aim of this systematic review is to answer the following questions: “Is there an effect of bleaching agents on incipient caries lesions?”; “Is there a difference in the effect of various types of bleaching agents on caries lesions?”; and ”What is the effect of several bleaching agents on biofilm adhesion and accumulation?”.

## 2. Materials and Methods

Cochrane’s protocol was followed in this review through Review Manager 5.4.1, the official software for Cochrane’s database [24]. Furthermore, the reporting of this review followed the recommendations of the latest PRISMA statement [25]. The present systematic review was registered on the PROSPERO platform (ID number CRD42023424805).

### 2.1. Search Strategy

This systematic review examines all studies that are focused on bleaching and its effects on healthy enamel or incipient caries, dividing all studies into five groups: (a) Bleaching and enamel demineralization, (b) Bleaching and bacterial adherence, (c) Bleaching and white spots/incipient caries/early caries (esthetic considerations), (d) Bleaching and white spots/incipient caries/early caries (mechanical considerations), (e) Bleaching agents and antimicrobial activity. The search strategy was based on the combination of the following terms: ((bleaching agents) OR (tooth bleaching) OR (bleaching) OR (tooth whitening) OR (in-office bleaching) OR (home bleaching) OR (carbamide peroxide) OR (hydrogen peroxide)) AND ((white spot lesion) OR (enamel demineralization) OR (early caries) OR (incipient caries) OR (caries) OR (tooth decay) OR (oral hygiene) OR (*S. mutans*) OR (*Streptococcus mutans*) OR (Lactobacilli) OR (Lactobacillus) OR (oral microbiome) OR (oral biofilm)). Two examiners (VK and RG) conducted the electronic search within published and unpublished literature separately. The primary formal databases utilized in this study were MEDLINE via Pubmed and Scopus. The language selected was English. The gray literature reports included theses, dissertations, product reports, and unpublished studies through Cochrane Central, Cochrane Database for Systematic Reviews, ClinicalTrials.com, Open Grey, and ISRCTN. No filters were used; the search was performed on 27 April 2023.

### 2.2. Inclusion Criteria

- The review included randomized controlled trials, clinical trials, in vitro studies, and observational studies;
- Studies comparing at least two groups;
- In vitro studies investigating the effect of tooth bleaching agents on the demineralization of enamel analyzed with a confocal laser scanning microscope and/or measured via enamel surface roughness, and studies measuring the concentration of cariogenic and periodontal microflora in the surrounding tissues, as well as by the presence of initial or advanced forms of caries;
- Studies assess the effect of bleaching on bacterial adherence to enamel;
- Studies examining the aesthetic effect of bleaching on white or brown spots of enamel and the mechanical effects of bleaching on early caries lesions;
- Studies investigating any effect of bleaching agents on the prevalence of caries (primary or secondary).

### 2.3. Exclusion Criteria

- Animal studies, case reports, and case series;
- Studies without at least one control and one test group, and studies without a comprehensive protocol;
- Studies with ineligible results for this review (i.e., studies that, when reading the full text, the results did not relate to this study);
- Lack of access to the full text of a study after attempting to contact the author.

### 2.4. Data Extraction

Data were extracted and recorded in standardized piloted forms (Zotero 5.0.47, Corporation for Digital Scholarship, and the Roy Rosenzweig Center for History and New Media). These forms include specific characteristics of the study (type, title, authors, abstract, publication, volume, issue, pages, date, series, series title, series text, journal abbreviation, language, DOI, URL, ISSN, short title, mean of access, archive, location in the archive, library catalog, call number, date added, date modified). Data were extracted by two of the reviewers (EP, GG) and re-examined by another two reviewers (VK, CR). Inconsistencies were discussed among reviewers until a consensus was reached.

### 2.5. Screening and Eligibility Check

The studies that were collected from all databases were cross-checked for the exclusion of duplicates. Titles and abstracts were screened independently by four reviewers (E.P., G.G., V.K., C.R.), according to the study’s main characteristics of interest, with further exploration of the full text. Each reviewer decided on the inclusion or exclusion of the studies according to eligibility criteria. Potential discrepancies were discussed among reviewers until a consensus was established.

### 2.6. Risk of Bias Assessment (RoB)

Cochrane Risk Of Bias tool 2.0 assessed the methodological quality of the studies for randomized controlled trials [26] and the ROBINS-I (Risk Of Bias In Non-randomized Studies—of Interventions) for controlled trials and observational studies [27]. Furthermore, the GRADE assessment tool was used to rate the strength of clinical recommendations [28].

In this manner, a configuration tool was developed to follow reporting, performance, and other types of bias. Each section consisted of preselected criteria to reveal poor data disclosure, encoded as “partly specified”, or total absence of examined data, encoded as “not specified”. The score “specified” encoded a complete and detailed description. The configuration tool was adjusted to enable assessment of the in vitro, in vivo, and systematic review studies to present comparable data in each category. Studies with unclear presentation of data were categorized as “partly specified”. 

Concerning the in vivo studies, the configuration tool underwent relevant modifications to apply specifically to the characteristics associated with this category. Based on the RoB 2 CRT tool, conversions were embedded to follow the requirements of the present systematic review. The domains followed were the Randomization Process, Deviations from Intended Interventions, Missing Outcome Data, Measurement of Outcome, and Reported Result. These sections consist of modified questions derived from the RoB 2 CRT tool, associated with the preselected criteria, and are met with encoded replies. These responses are PY (probably yes), Y (yes), PN (probably not), N (not), and NA (not available). 

## 3. Results

**Figure 1:**
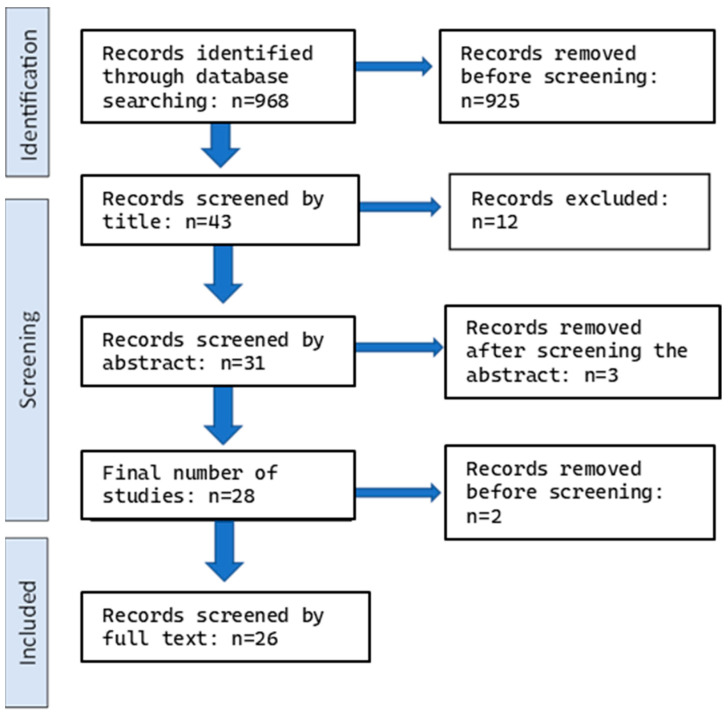
Flow chart of the study, showing number of studies during identification and screening.

Figure 1 shows the flow chart of this study.

Table 1 presents the categorization of selected studies according to their type.

Out of 968 unique records (filtered using an electronic database and hand searches), 28 studies were left for a full-text evaluation by the qualifying criteria. 

The principal categorization led to two domains: the in vitro and the in vivo studies. The first division included all types of in vitro studies, counting 23 in total, which underwent further classification, as can be viewed in Table 1.

Study characteristics and qualitative data are detailed in Table 2, Table 3 and Table 4. The first cluster (n = 7) has been generated to identify studies that included teeth and microbial factors [29,30,31,32,33,34,35]. The second cluster (n = 12) contained studies involving teeth that did not use microbial cultivation [36,37,38,39,40,41,42,43,44,45,46,47]. The third cluster (n = 4) included studies that did not involve either microbial or dental tissue [48,49,50,51]. The fourth cluster included clinical studies [52,53,54,55,56]. 

In the first cluster (Table 2), one study showed an increase in adhesion of *S. mutans* [29] after bleaching whilst others showed a decrease in adhesion of *S. mutans* and biofilm thickness [30,31], or else no difference [33] or at least not an increase in caries susceptibility [34]. Nevertheless, the study that showed no difference in *S. mutans* also showed a change in enamel roughness and an increase in *S. sanguis* adhesion [33]. For the enamel structure, these studies showed that, with or without laser treatment, in addition to bleaching, there was alteration in enamel microstructure [31], but, when plasma light treatment was performed, there was not an alteration in enamel microstructure [35].

In the second cluster (Table 3), one study showed that using 10% carbamide peroxide on white spots decreases color disparities without an alteration in mineral composition [36], but another study showed an increase in the demineralization depth of those lesions [38]. One more study showed that bleaching procedures lead to caries arresting [39], and the studies from Alves et al. and Pretty et al. showed that there is no effect from bleaching on caries [45,47]. Two more studies showed that there is a mineral loss from sound enamel during bleaching but not from carious enamel [40,41]. Concerning the masking effect, one study concluded that there is no sound masking of white spots [43] and two others concluded that there is adequate masking [39,44] and that the masking is better when performing at-home bleaching rather than in-office [44].

Three of the in vitro studies in cluster 3 (Table 4) concluded that there is an increase in adhesion of bacterial biofilm during bleaching procedures using different composites [48,49,50]. Nevertheless, the fourth study in this cluster showed a higher antibacterial effect of bleaching than chlorhexidine [51].

The fourth cluster contained all the in vivo studies (Table 5). Three of them concluded that there were no alterations in bacteria counts after bleaching procedures (in saliva, buccal mucosa, and plaque) [53,54,55], whilst one of them showed a decrease in *S. mutans* in plaque and saliva after bleaching [52]. The in vivo study concerning the masking of white spots after bleaching concluded that there is adequate masking [56].

### 3.1. Results of Risk of Bias Assessment

**Figure 2:**
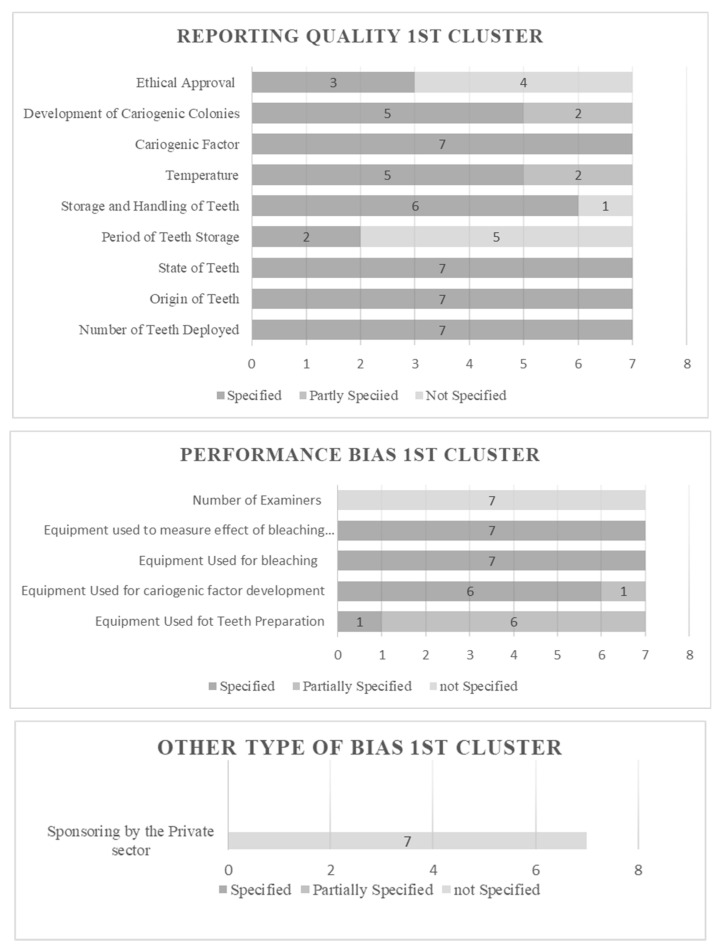
Risk of Bias evaluative data for cluster 1.

**Figure 3:**
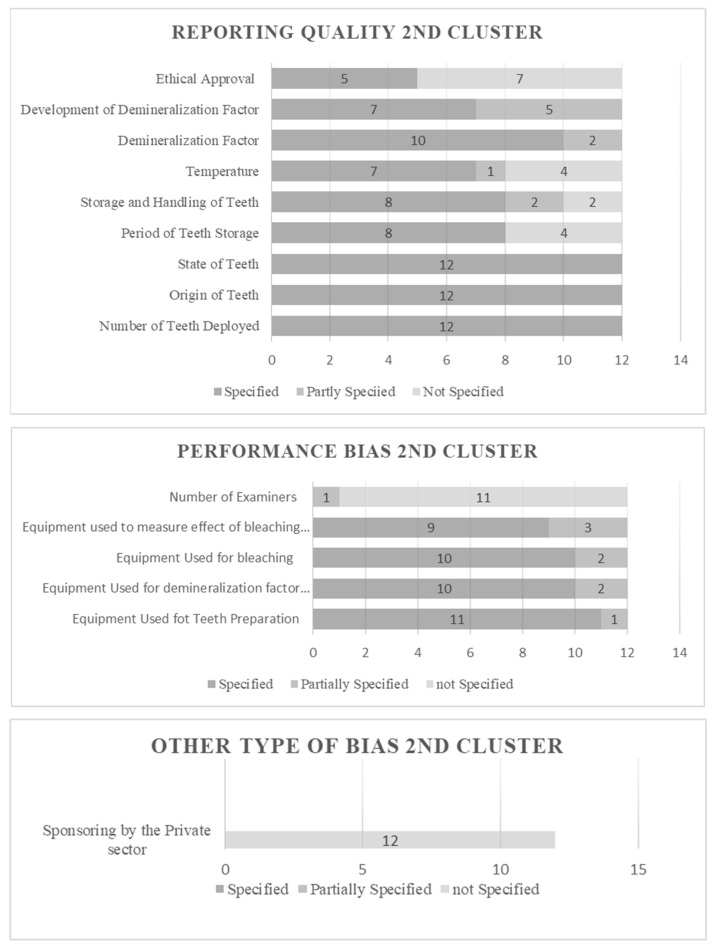
Risk of Bias evaluative data for cluster 2.

**Figure 4:**
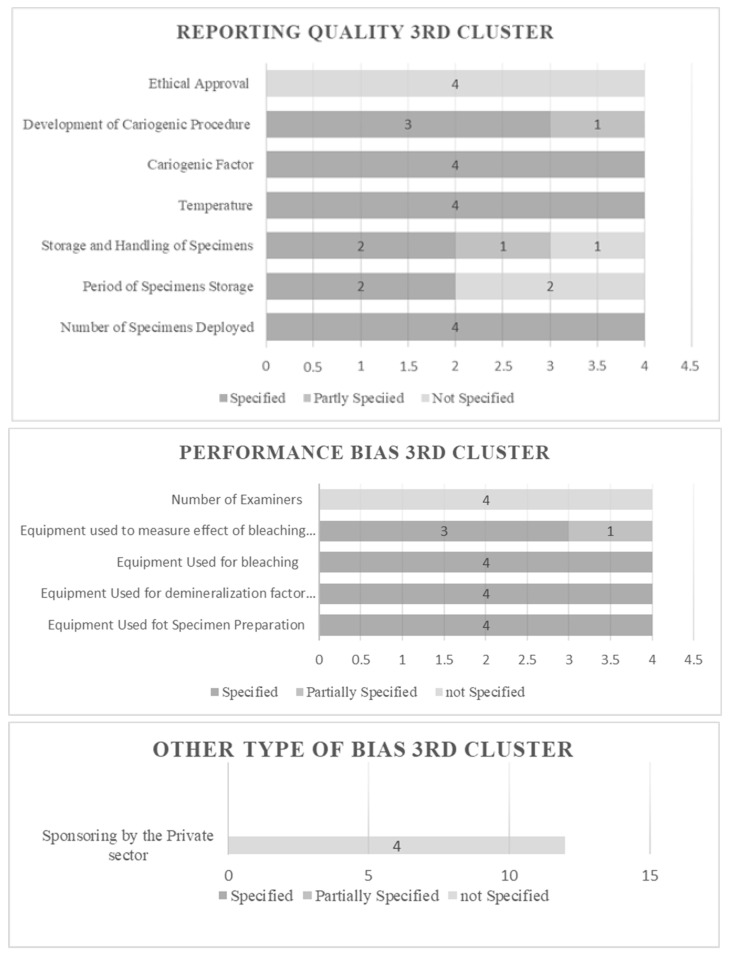
Risk of Bias evaluative data for cluster 3.

**Figure 5:**
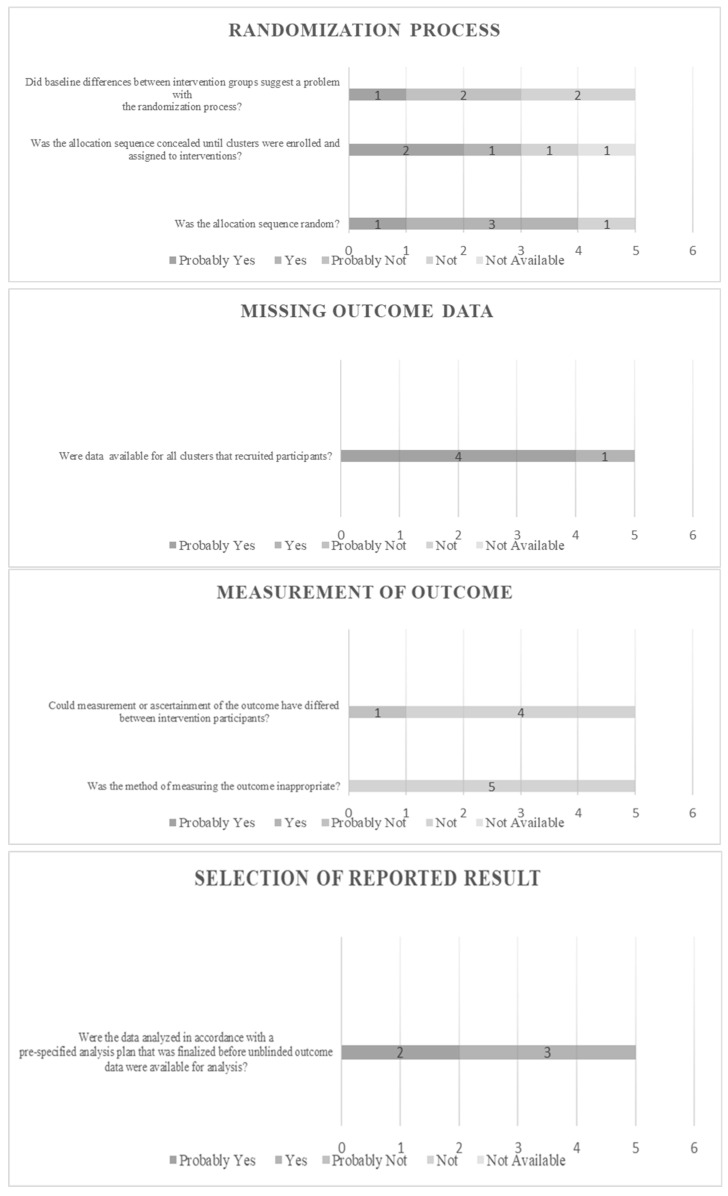
Risk of Bias evaluating data for clinical studies.

The risk of bias in individual studies was assessed based on fixed categorization relative to the design. The selected studies were enumerated, categorized, and estimated. All features extracted under the established principle fulfill the predetermined criteria. The characteristics were objectified and presented in the form of evaluative data. The results can be viewed in Figure 2, Figure 3, Figure 4 and Figure 5 for Clusters 1–4. 

In total, seven studies were classified as cluster 1, which was dispositioned based on the deployment of teeth and bacteria. In the reporting quality, all studies provided data on the number, state, and origin of teeth, while 28.6% (two) specified the period of tooth storage. Regarding storage and handling of teeth, 85.7% (six) of the studies provided fully specified data. Temperature, a factor that is occasionally omitted, was partially specified in two (28.6%) out of seven studies. The approach of bacterial identity was fully specified, and the development procedure was partially specified in 28.6% (two) of the studies, revealing a relatively high precision comparatively or proportionally to the one followed in tooth identification, preparation, and handling. A relatively high proportion, 57.1% (four), of the studies needed to provide specifications for ethical approval by a committee. The performance bias tabloid examination showcased that six out of seven studies provided a partially specified description of the equipment used for tooth preparation, equivalent to 85.7%. In contrast, only one study provided a fully specified description. The reverse result was observed for the equipment specification deployed for the cariogenic factor development, leading to 85.7% of the studies demonstrating a detailed presentation of the relevant data. Concerning the equipment used for bleaching and for measuring the effect of bleaching, all studies presented with complete specifications. Finally, in the first cluster, a study must fully specify the number of examiners participating in these actions. In the “other type of bias” section, no study provided full specifications of the participation of third-party sponsors. 

Figure 3 demonstrates the results associated with the “no bacteria involved” cluster. In total, 12 studies were categorized as such, and all contained specified data concerning the number, origin, and state of teeth deployed. Concerning the period of tooth storage, 33.3% (four) of the studies did not provide specified data, while, when examining the storage and handling of teeth, 16.7% (two) of the studies did not provide specified data, and 16.7% of the studies provided partially specified data. The investigation or temperature report revealed that 4 out of 12 studies (33.3%) did not provide specified data, and 8.3% (1 study) provided partially specified data. Regarding the demineralization factor, 10 studies (83.3%) contained specified data, and 2 (16.7%) provided partially specified data. Disclosure of the “development of demineralization factor” data led to seven studies providing specified ones (58.3%) and five (41.7%) provided partially specified. Lastly, an examination of the report of ethical approval revealed that seven studies did not provide specified data (58.3%) and five did (41.7%). In the “performance bias” section, when scrutinizing the equipment used for tooth preparation, 11 studies (91.7%) specified it, and 1 (8.3%) provided partially specified data. Examining the equipment used for the demineralization factor, 83.3% (10) of the studies produced specified data, and 16.7% (2) partially specified. When scrutinizing the equipment used for bleaching, the same results were produced as those collected in the “equipment used for the demineralization factor”. Additionally, when investigating the equipment used to measure the effect of bleaching, nine studies (75%) were specified, and three (25%) were partially specified. Finally, when investigating the number of examiners, results revealed that 11 studies (91.7%) did not specify the number of scrutineers, while 1 study provided a partial specification (8.3%) of their number, as it did not identify the number of examiners in every stage. In the “other type of bias” section, all 12 studies did not provide specified data concerning third-party sponsorship.

Figure 4 presents the results of studies that did not involve the deployment of teeth. The reporting quality configuration includes all four identified studies that disclosed specified data concerning the number of specimens deployed. Examining the period of specimens’ storage, 50% did not specify data, and 50% provided full specifications. In storing and handling specimens, two studies provided specified data, one study provided partially specified, and one did not specify (50%, 25%, and 25%, respectively). All studies fully specified temperature, which resulted in examining the cariogenic factor and scrutinizing the development of cariogenic procedures; this led to the revelation that 75% of the studies contained fully specified data, and 25% contained partially specified. Finally, when acquiring ethical approval, all four studies did not provide specific data. The performance bias section concerning the equipment used for specimen preparation, the demineralization factor, and the one used for bleaching contained specified data for all studies. Examining the equipment used to measure the effect of bleaching on lesions resulted in 75% of the studies providing specified data and 25% providing partially specified. Finally, no study disclosed data on the number of examiners performing measurements, and the same outcome stood for the “other type of bias”.

In Figure 5, the in vivo studies are clustered and assessed. Four categories were configured. In this section, according to the PRISMA and RoB2 principles, the scoring was formed as “probably yes”, “yes”, “probably not”, “not”, and “not available”, depending on the data derived from the study evaluation. In the “randomization process” assessment, three major subcategories were scrutinized. When focusing on possible differences between intervention groups that could be associated with problems in randomization, one study (20%) scored “possible yes”, two studies scored “yes” (40%), and the same score was obtained for the “probably not” selection. Concerning the question about the allocation and whether it was concealed until the clusters were assigned and enrolled in the interventions, two (40%) of the studies scored “probably yes”, one (20%) scored “yes”, one (20%) scored “probably not”, and one (20%) scored “not”. Finally, in the question about the randomness of the allocation sequence, one (20%) study scored “possibly yes”, three (60%) scored “yes”, and one (20%) scored “probably not”. In the “missing outcome data” section, one question was formed for this review to examine the availability of data for all clusters that recruited participants. The results revealed that four (80%) of the studies scored “probably yes” and one (20%) scored “yes”. Assessment of the “measurement of outcome” was performed by two major inquiries. When scrutinizing the “method of measuring the outcome and its possible impropriety,” all studies scored “possibly not”. On the question “Could measurement or ascertainment of the outcome have differed between intervention participants?”, four (80%) studies scored “probably not” and one (20%) scored “yes”. Finally, in the section “selection of reported result,” the inquiry examined was whether “the data were analyzed in accordance with a pre-specified analysis plan that was finalized before unblinded outcome data were available for analysis,” which led to the revelation that two (40%) studies scored “possibly yes” and three (60%) scored “yes”. 

### 3.2. Metanalysis

Initially, all studies were examined to verify the providence of quantitative data. The outcome of the initial examination revealed that all the investigated studies displayed data in qualitative form. Parts of the data were displayed in tables. These parts were in quantitative form, leading to the revelation that 14.3% (four) presented tables with microhardness measurements performed using various techniques. Two studies (7.1%) demonstrated numeric data related to mineral loss on measured surfaces, and another 7.1% (two) of the studies showed means and SDs in color changes. 

The nature of these data, along with studies that did not proceed in an analogous manner, led the researchers to contact the investigators, aiming to acquire full access to quantitative data as the sole path to enabling meta-analysis calculations. 

From all 28 studies, 20 corresponding authors did not reply at all (71.4%), 2 studies did not provide any contact details (7.1%), 3 corresponding authors replied and provided quantitative data (10.7%), and 3 studies had provided invalid corresponding emails (10.7%). Thus, due to the lack of information and the heterogeneity of the data, the meta-analysis could not be performed.

Assessing these outcomes, it was unanimously decided by the conductors of this systematic review to not proceed with the meta-analysis since the existing quantitative data would not cover the multilateral conclusions from the various clusters examined in the systemic review. 

## 4. Discussion

The dental society is intrigued by tooth whitening’s effect on cariogenic activity, and this systematic review attempted to shed light on the methodological approaches. 

The number of studies selected for this systematic review fulfills several criteria, which were deployed under the realization that such diversity in approaches and protocol-based regimes would require multiple configuration tools. Performing a casual control on the outcomes of the studies resulted in 6 (21.4%) supporting increased attachment capacity and cariogenic dynamics, 4 (14.3%) supporting decreased adhesion and cariogenic activity, 7 (25%) showing no difference, and 11 (39.3%) approaching the subject in a variated manner to such extent that they could not be categorized in this fashion. Furthermore, 39.3% of the studies deployed an abundance of variations; thus, organizing them in subgroups necessitated installing a strictly produced tool. Moreover, some studies, e.g., “Effect of salivary biofilm on the adherence of oral bacteria to bleached and non-bleached restorative material”, generated an outcome that described differentiated behavior between examined strains of bacteria, a fact that forged skepticism on the appropriate categorization and assessment of such studies [49].

On the other hand, a wide range of methods of investigation was detected. While the primary goal of this review was to produce results based on bias assessment protocols, a fundamental restriction is the fact that the scientific community approaches the subject with a wide range of studies. This fact created challenges when attempting to configure an objective mechanism of assessment. The existing data concerning the performance of bleaching agents and etching materials create ambiguity, especially regarding the performance of dental tissues and cariogenic factors. At first glance, the number of studies that resulted in increased cariogenic activity and, on the opposite side, the ones that concluded decreased cariogenic dynamics consists of 35.7% of the total number of studies, which indicates that further research is required to reach more solid outcomes.

Additionally, almost all in vitro studies (22 out of 23), regardless of the cluster in which they were categorized, did not provide data concerning the number of examiners, and none of the 23 studies specified sponsorship by private initiatives. This element comes as a response to the fact that some studies examine commercial products, e.g., “Antibacterial Activity of 10% Carbamide Peroxide Bleaching Agents” [56], but did not provide data for possible private sponsorship or any funding sources. 

These factors indicate that certain elements of the studies included in this systematic review, as revealed by the investigative procedure, present a tendency towards bias when examiners focus on commercial products (bleaching agents). 

The plethora of individual features examined in the three clusters of the in vitro studies showcased that, although the overall strategy is articulated, certain areas imply a tendency towards only a weak demonstration of qualitative data. When it comes to the storage of the samples and handling in the reporting quality section, 52.2% of all in vitro studies provided specified data, 13% partially specified, and 34.8% did specify, while, regarding the period of storage, 52.2% demonstrated specified data and 47.8% did not specify. These features indicate a high proportion of possible bias and are mentioned as a part of the revelation of specific zones of weakness. 

This systematic review aims to highlight the need for a scientifically documented and universally accepted practice and not simply follow clinical instructions by experts and established institutions.

In vivo studies, on the other hand, were thoroughly examined and revealed important data. When examining the allocation and randomization process, the percentage of those studies with possible bias due to lack of demonstrated specifications was scarce. Judging based on the PRISMA guidelines, the conclusion calls for the requirement of further established formalism when scientific research is performed at the clinical level within the field of dentistry. Apart from this, in general, the in vivo studies performed satisfactorily. 

More specifically, when focusing on the studies that demonstrated an increase in bacterial adhesion, a study from 2003 concluded that agglutination expanded at a minimum up to 500% [29], and another one from 2015 assumed that the bleaching demineralized enamel and altered the microhardness values, which showed more pronounced changes than the group that was not bleached to a depth of 90 μm [38]. On the other hand, a study from 2009 found that carbamide peroxide bleaching promoted enamel demineralization [40]. Similarly, another study in their results highlighted that those bleaching treatments increased enamel demineralization, while Ca ions or ACP could not alter the path of the process [41]. Similarly, a different study concluded that bleaching with 10% CP or 40% HP increased *S. mutans* and *S. sanguinis* biofilm formation on composite resin restorations, while enamel surface roughness also seemed to be increased [50]. 

On the opposite side, a study from 2011 showed that the plaque indexes were significantly lower than those before bleaching, with TPI remaining lower than that of the baseline, even in the second week after bleaching, a fact that showcased the decrease in the microbial activity [52]. A different one from 2016 showed that the 10% carbamide peroxide bleaching of enamel with white spot lesions increased esthetic results and did not only cause deterioration of the mineral composition, but also remineralized the subsurface body lesion [36]. In the same way, another study led to the result that bleaching could significantly increase enamel surface roughness but inhibit the formation of biofilms of *S. mutans* until three days after the application of bleaching [30]. 

The demonstration of results that are conflicting raises the requirement for further investigation. This effort could produce a greater quantification of these conflicting results to arrive at more definitive conclusions. As it stands, based on the numerous studies examined in this systematic analysis, it cannot be supported that bleaching plays an aggravating or positive role in white spot lesions, dental tissues, and restoration materials, nor in microbial colonies.

To the best of our knowledge, this is the first comprehensive systematic review of in vitro and in vivo studies assessing the effects of bleaching agents on carious and biofilm status on hard tissues. Some limitations exist. A high number of the studies were characterized as having a high risk of bias. Overall, due to the wide variation in methods used in the investigations and the variability in results obtained, a meta-analysis could not be performed and no firm conclusions could be stated. 

## 5. Conclusions

Conflicting outcomes were observed as to the impact of bleaching agents and methods on incipient caries and biofilm. The majority of the studies presented a high risk of bias. This systematic review, recognizing the limitations associated with a scientific survey of studies that incorporate diverse methodologies, has led to the disclosure of some methodological areas that are prone to bias. It remains to not only compare studies per se, but to investigate methodological gray zones and, thus, demonstrate the necessity of a more meticulous and precise organization of the staging of the scientific steps. A meta-analysis can then be performed to investigate the outcomes further. Nevertheless, as far as carbamide peroxide bleaching is concerned, there does not seem to be a clinically significant alteration in microorganism counts nor in enamel microstructure.

## Figures and Tables

**Table 1:** Categorization of selected studies following their type and cluster classification.

In Vitro		23
Cluster 1 (Bacterial adherence on teeth)	7	
Cluster 2 (No bacteria involved)	12	
Cluster 3 (No teeth deployment)	4	
Clinical (Cluster 4)		5
Total number of studies		28

**Table 2:** Cluster 1: Study characteristics and qualitative data.

	Study	Aim	Design	Sample Size and Basic Features	Findings
**1.**	“Changes in enamel surface roughness and adhesion of *Streptococcus mutans* to enamel after vital bleaching” Hosoya et al., 2003 [29]	To observe the influence of vital bleaching on the enamel surface and adhesion of *S. mutans* to tooth enamel.	In vitro	- 70 fresh human impacted third molars; - 50 mL of pre-cultivated *S. mutans*.	Teeth displayed increased adhesion of *S. mutans* colonies.
**2.**	“Effects of Cold-Light Bleaching on Enamel Surface and Adhesion of *Streptococcus mutans*” Zhang et al., 2021 [30]	To investigate how cold-light bleaching influences enamel roughness and adhesion of *S. mutans.*	In vitro	24 maxillary premolars, extracted for orthodontic purposes.	- Enamel surface roughness increased after bleaching; - The adhesion of *S. mutans* was decreased on bleached enamel in a turbidity test; - The thickness of biofilms was decreased on bleached enamel; - *S. mutans* was less inclined to adhere to bleached enamel based one analysis by CLSM (confocal laser scanning microscopy).
**3.**	“Effects of Bleaching Associated with Er:YAG and Nd:YAG Laser on Enamel Structure and Bacterial Biofilm Formation” Hou et al., 2021 [31]	To compare the effects of bleaching associated with Er:YAG and Nd:YAG lasers on enamel structure and mixed biofilm formation on tooth surfaces.	In vitro	68 enamel samples were prepared from sound permanent third molars.	The enamel surface structure significantly changed after bleaching both with and without laser treatment.
**4.**	“Antimicrobial Activity and the Effect of Green Tea Experimental Gels on Teeth Surfaces” Voina et al., 2020 [32]	To evaluate an experimental green tea extract and an experimental green tea gel for enamel-restoring treatment after bleaching. To test the antibacterial and antifungal effect of the experimental extract against specific microorganisms.	In vitro	28 healthy third molars, extracted for orthodontic purposes.	- The green tea extract solution resulted in an important antibacterial effect on *P. anaerobius* and *S. mutans strains*; - The natural extract based on Camellia sinensis has antimicrobial activity; - The extract has no antifungal action on the *C. albicans* strain.
**5.**	“In-office bleaching gel with 35% hydrogen peroxide enhanced biofilm formation of early colonizing streptococci on human enamel” Ittatirut et al., 2014 [33]	To compare the effects of 25% and 35% hydrogen peroxide in-office bleaching systems on surface roughness and streptococcal biofilm formation on human enamel.	In vitro	162 enamel specimens were prepared from sound permanent anterior and premolar teeth.	- Bleaching with both 25% and 35% H_2_O_2_ systems significantly decreased the enamel surface roughness comparing to the control; - *S. sanguis* biofilm formation on enamel bleached with 35% H_2_O_2_ was markedly higher; - No significant difference in *S. mutans*.
**6.**	“The Effect of Whitening Agents on Caries Susceptibility of Human Enamel” Al-Qunaian et al., 2005 [34]	Evaluated whether the treatment of human enamel with whitening agents containing different concentrations of carbamide or hydrogen peroxide changed the susceptibility of enamel to caries.	In vitro	- 24 sound human incisors were selected; - 3 groups were created.	Application of bleaching agents does not increase caries susceptibility of human enamel. Also, a bleaching agent-containing fluoride reduced caries susceptibility.
**7.**	“Tooth bleaching with low temperature plasma lowers surface roughness and *Streptococcus mutans* adhesion” Nam et al., 2017 [35]	To evaluate the structural–morphological changes in enamel surface roughness and *S. mutans* adhesion after tooth bleaching with plasma activation in combination with a low concentration of 15% carbamide peroxide.	In vitro	60 single-rooted human premolars with intact crowns extracted for orthodontic reasons.	The plasma activation did not cause changes in enamel. Combination methods using plasma and low concentrations of 15% carbamide peroxide caused even smaller changes in enamel.

**Table 3:** Cluster 2: Study characteristics and qualitative data.

	Study	Aim	Design	Sample Size and Basic Features	Findings
**1.**	“Bleaching Effects on Color, Chemical, and Mechanical Properties of White Spot Lesions” Kim et al., 2016 [36]	To evaluate the effect of bleaching on teeth with white spot lesions.	In vitro	20 human upper premolars extracted during dental treatments were used.	10% carbamide peroxide of enamel with white spots minimized color disparities without altering mineral composition or microhardness. CPP–ACP remineralized the subsurface body lesion.
**2.**	“Bleaching of simulated stained-remineralized caries lesions in vitro” Angari et al., 2018 [37]	To test the efficacy of bleaching on demineralized white spot lesion esthetic treatment.	In vitro	Enamel and dentin samples were sectioned from the buccal and lingual surfaces of human molars.	Stains that are nonmetallic respond better to bleaching.
**3.**	“Demineralization and Hydrogen Peroxide Penetration in Teeth with Incipient Lesions” Briso et al., 2015 [38]	To evaluate the demineralization and hydrogen peroxide penetration in teeth with incipient lesions submitted to bleaching treatment.	In vitro	100 sound permanent bovine incisors obtained from steers aged between 24 and 30 months were selected.	The bleaching treatment can increase the demineralization depth of incipient carious lesions.
**4.**	“Do bleaching gels affect the stability of the masking and caries-arresting effects of caries infiltration—in vitro” Jansen et al., 2020 [39]	To evaluate the influence of different bleaching gels on the masking and arresting caries of infiltrated and non-infiltrated stained artificial enamel caries lesions.	In vitro	Bovine incisors were extracted from freshly slaughtered cattle (negative BSE test), cleaned, and preserved in 0.08% thymol. The teeth were separated in 400 enamel blocks.	The bleaching agents could improve the esthetic appearance of infiltrated and non-infiltrated stained enamel lesions.
**5.**	“Effect of 10% Carbamide Peroxide Bleaching on Sound and Artificial Enamel Carious Lesions” Pinto et al., 2009 [40]	To evaluate the effect of 10% carbamide peroxide bleaching on Knoop surface microhardness and morphology of sound enamel and enamel with incipient caries after pH-cycling.	In vitro	40 blocks with known surface microhardness were selected, made of extracted human third molars that had been erupted.	Carbamide peroxide bleaching demineralized sound enamel but not carious enamel.
**6.**	“Effect of Bleaching on Sound Enamel and with Early Artificial Caries Lesions Using Confocal Laser Microscopy” Berger et al., 2012 [41]	To evaluate the effect of bleaching agents on sound enamel and incipient caries using confocal laser scanning microscopy.	In vitro	20 extracted sound bovine incisors stored in 0.1% thymol solution, from which 80 (4 × 5 × 5 mm) samples of enamel were formed.	The bleaching treatments increased sound enamel demineralization, while the addition of Ca ions or ACP did not cause any changes. Incipient caries were not affected.
**7.**	“In vitro demineralization of tooth enamel subjected to two whitening regimens” Ogura et al., 2013 [42]	To examine the level of demineralization of human tooth enamel after home or in-office bleaching with photo activation.	In vitro	30 human premolars without stain or defect, extracted for orthodontic purposes.	In-office was favorable, according to demineralization.
**8.**	“Investigation of the Esthetic Outcomes of White Spot Lesion Treatments” Lee 2022 [43]	To compare the ability of bleaching, resin infiltration, and microabrasion to mask existing white spot lesions (WSL).	In vitro	Extracted human maxillary incisor teeth—only teeth with WSLs were selected.	Among the three investigated treatment modalities, resin infiltration was most able to mask WSLs.
**9** **.**	“Dental bleaching efficacy and impact on demineralization susceptibility of simulated stained-remineralized caries lesions” Al-Angari et al., 2018 [44]	To evaluate the color improvement of stained remineralized carious lesions with different bleaching systems and to evaluate the susceptibility of the bleached lesions to further demineralization.	In vitro	Human enamel samples (n = 21 per group).	At-home bleaching (15% CP) results in better esthetic results.
**10.**	“Susceptibility to caries like lesions after dental bleaching with different techniques” Alves et al., 2007 [45]	To evaluate the effect of dental bleaching on the susceptibility of developing caries-like lesions.	In vitro	30 unerupted human third molars.	- In-office dental bleaching 37% CP (halogen light); - 35% HP (LED light) does not influence caries-like lesion development; - Home bleaching 10% and 16% CP with 0.2% sodium fluoride reduces susceptibility.
**11.**	“The ability of dual whitening anti-caries mouthrinses to remove extrinsic staining and enhance caries lesion remineralization—An in vitro study” Al-Shahrani et al., 2020 [46]	To investigate the ability of these mouthrinses to remove staining from artificially stained carious lesions and to improve remineralization.	In vitro	- Bovine enamel specimens; - Teeth crowns were cut into 4 × 4 mm^2^. - A total of 250 specimens were prepared.	Artificially stained enamel caries lesions show reduced susceptibility to fluoride remineralization and whitening effects of commercial whitening and anti-caries mouthrinses.
**12.**	“The effect of bleaching on enamel susceptibility to acid erosion and demineralization“ Pretty et al., 2005 [47]	To determine if bleaching with carbamide peroxide increases the risk of erosion or demineralization.	In vitro	24 human incisors selected with specified criteria.	Tooth bleaching with carbamide (urea) peroxide (using commercially available concentrations) does not increase risk of acid erosion or caries.

**Table 4:** Cluster 3: Study characteristics and qualitative data.

	Study	Aim	Design	Sample Size and Basic Features	Findings
**1.**	“Bacterial adherence to bleached surfaces of composite resin in vitro” Mor et al., 1998 [48]	To examine the effect of bleaching on bacterial adherence to composite resin restorations.	In vitro	162 samples of Polofil Supra light-curing resin-based composite (Voco, Cuxhaven, Germany).	Carbamide peroxide and hydrogen peroxide can alter the adherence of microorganisms to polished surfaces of composite resin restorations.
**2.**	“Effect of salivary biofilm on the adherence of oral bacteria to bleached and non-bleached restorative material” Steinberg et al., 1999 [49]	To examine the effect of in vitro biofilm on adherence of microorganisms on bleached and non-bleached restorative materials.	In vitro	162 samples of Charisma Supra-light-curing resin-based composite.	Salivary biofilm on bleached restorations altered the adhesion of *S. mutans*, *S. sobrinus*, and *A. viscosus*.
**3.**	“Effect of Vital Tooth Bleaching on Surface Roughness and Streptococcal Biofilm Formation on Direct Tooth-Colored Restorative Materials” Wongpraparatana et al., 2017 [50]	To compare the effect of bleaching with a 10% carbamide peroxide or a 40% hydrogen peroxide system on surface roughness of resin composite and resin-modified glass ionomer cement (RMGI) and the formation of biofilm.	In vitro	2 restorative materials (a nanofilled resin composite material and an RMGI). 108 samples of each material in shade A2 were formed as disks of 5 mm in diameter and 2 mm thickness.	The bleaching systems used, 10% CP or 40% HP, significantly increased both the surface roughness and the streptococcal biofilm formation on resin composite and resin-modified glass ionomer cement.
**4.**	“Antibacterial Activity of 10% Carbamide Peroxide Bleaching Agents” Gurgan et al., 1996 [51]	To examine the antibacterial properties of 3 carbamide peroxide bleaching agents (Nite White, Karisma, and Opalescence) on *S. mutans, S. mitis, S. sanguis, Lactobacillus casei,* and *Lactobacillus acidophilus.*	In vitro	*S. mutans* (type A, 10,919), *S. mitis* (type A, 4a), *S. sanguis* (type A, 6b), *Lactobacillus casei* (type 319), and *Lactobacillus acidophilus* (type A, 161).	All bleaching materials showed significant antibacterial effect when compared to 0.2% chlorhexidine solution.

**Table 5:** Study characteristics and qualitative data (cluster 4).

	Study	Aim	Design	Sample Size and Basic Features	Findings
**1.**	“Effects of Hydrogen Peroxide-Containing Bleaching on Cariogenic Bacteria and Plaque Accumulation” Zheng et al., 2011 [52]	To evaluate the effects of a bleaching agent containing 36% hydrogen peroxide on the plaque index and *S. mutans* and Lactobacilli counts in dental plaque and saliva.	In vivo/clinical	20 medically fit adult volunteers.	*S. mutans* decreased significantly in plaque and saliva 4 weeks after bleaching.
**2.**	“A clinical, randomized study on the influence of dental whitening on *Streptococcus mutans* population” Briso et al., 2017 [53]	To evaluate the influence of home bleaching on *S. mutans* counts in saliva, buccal mucosa, and plaque.	Clinical/in vivo	30 individuals were selected, meeting the criteria for inclusion.	No change was observed when whitening was performed with carbamide peroxide.
**3.**	“Comparative Study of the Effects of Two Bleaching Agents on Oral Microbiota” Alkmin et al., 2005 [54]	To evaluate the effects of bleaching with 10% carbamide peroxide (Platinum/Colgate) or 7.5% hydrogen peroxide (Day White 2Z/Discus Dental) on *S. mutans.*	In vivo	30 volunteers who needed dental bleaching.	No changes were found in the *S. mutans* counts during bleaching treatment.
**4.**	“External Bleaching Effect on the Color and Luminosity of Inactive White-Spot Lesions after Fixed Orthodontic Appliances” Knosel et al., 2007 [55]	To evaluate the effect of bleaching on the color and luminosity of inactive white spot lesions (WSLs) after orthodontic treatment.	In vivo/clinical	10 patients with WSLs after orthodontic treatment.	External bleaching can satisfactorily mask WSLs.
**5.**	“The effect of combined bleaching techniques on oral Microbiota” Franz-Montan et al., 2009 [56]	To evaluate the antimicrobial activity of 10% and 37% carbamide peroxide during 3 different techniques of dental bleaching.	In vivo	32 patients assigned to 4 groups.	Carbamide peroxide when used at 37%, 10%, or in combination does not affect salivary microorganisms.

## Data Availability

The raw data supporting the conclusions of this article will be made available by the authors on request.

## References

[1] Kothari S., Gray A.R., Lyons K., Tan X.W., Brunton P.A. (2019). Vital bleaching and oral-health-related quality of life in adults: A systematic review and meta-analysis. J. Dent..

[2] Al-Zarea B.K. (2013). Satisfaction with appearance and the desired treatment to improve aesthetics. Int. J. Dent..

[3] Fernández E., Bersezio C., Bottner J., Avalos F., Godoy I., Inda D., Vildósola P., Saad J., Oliveira O.B., Martín J. (2017). Longevity, esthetic perception, and psychosocial impact of teeth bleaching by low (6%) hydrogen peroxide concentration for in-office treatment: A randomized clinical trial. Oper. Dent..

[4] Geus J.L., de Lara M.B., Hanzen T.A., Fernández E., Loguercio A.D., Kossatz S., Reis A. (2015). One-year follow-up of at-home bleaching in smokers before and after dental prophylaxis. J. Dent..

[5] Moncada G., Sepúlveda D., Elphick K., Contente M., Estay J., Bahamondes V., Fernandez E., Oliveira O.B., Martin J. (2013). Effects of light activation, agent concentration, and tooth thickness on dental sensitivity after bleaching. Oper. Dent..

[6] Tay L.Y., Kose C., Loguercio A.D., Reis A. (2009). Assessing the effect of a desensitizing agent used before in-office tooth bleaching. J. Am. Dent. Assoc..

[7] Kina J.F., Huck C., Riehl H., Martinez T.C., Sacono N.T., Ribeiro A.P., Costa C.A. (2010). Response of human pulps after professionally applied vital tooth bleaching. Int. Endod. J..

[8] Soares D.G., Basso F.G., Hebling J., de Souza Costa C.A. (2014). Concentrations of and application protocols for hydrogen peroxide bleaching gels: Effects on pulp cell viability and whitening efficacy. J. Dent..

[9] Soares D.G., Basso F.G., Pontes E.C., Garcia Lda F., Hebling J., de Souza Costa C.A. (2014). Effective tooth-bleaching protocols capable of reducing H2O2 diffusion through enamel and dentine. J. Dent..

[10] Paula E.A., Kossatz S., Fernandes D., Loguercio A.D., Reis A. (2014). Administration of ascorbic acid to prevent bleaching-induced tooth sensitivity: A randomized triple-blind clinical trial. Oper. Dent..

[11] Rezende M., Loguercio A.D., Kossatz S., Reis A. (2016). Predictive factors on the efficacy and risk /intensity of tooth sensitivity of dental bleaching: A multi regression and logistic analysis. J. Dent..

[12] de Geus J.L., Wambier L.M., Kossatz S., Loguercio A.D., Reis A. (2016). At-home vs In-office Bleaching: A Systematic Review and Meta-analysis. Oper. Dent..

[13] Maran B.M., Burey A., de Paris Matos T., Loguercio A.D., Reis A. (2018). In-office dental bleaching with light vs. without light: A systematic review and meta-analysis. J. Dent..

[14] Maran B.M., Matos T.P., de Castro A.D.S., Vochikovski L., Amadori A.L., Loguercio A.D., Reis A., Berger S.B. (2020). In-office bleaching with low/medium vs. high concentrate hydrogen peroxide: A systematic review and meta-analysis. J. Dent..

[15] Luque-Martinez I., Reis A., Schroeder M., Muñoz M.A., Loguercio A.D., Masterson D., Maia L.C. (2016). Comparison of efficacy of tray-delivered carbamide and hydrogen peroxide for at-home bleaching: A systematic review and meta-analysis. Clin. Oral. Investig..

[16] Zanolla J., Marques A., da Costa D.C., de Souza A.S., Coutinho M. (2017). Influence of tooth bleaching on dental enamel microhardness: A systematic review and meta-analysis. Aust. Dent. J..

[17] Höchli D., Hersberger-Zurfluh M., Papageorgiou S.N., Eliades T. (2017). Interventions for orthodontically induced white spot lesions: A systematic review and meta-analysis. Eur. J. Orthod..

[18] Bourouni S., Dritsas K., Kloukos D., Wierichs R.J. (2021). Efficacy of resin infiltration to mask post-orthodontic or non-post-orthodontic white spot lesions or fluorosis—A systematic review and meta-analysis. Clin. Oral. Investig..

[19] Hayashi M., Momoi Y., Fujitani M., Fukushima M., Imazato S., Kitasako Y., Kubo S., Nakashima S., Nikaido T., Shimizu A. (2020). Evidence-based consensus for treating incipient enamel caries in adults by non-invasive methods: Recommendations by GRADE guideline. Jpn. Dent. Sci. Rev..

[20] Attin T., Schmidlin P.R., Wegehaupt F., Wiegand A. (2009). Influence of study design on the impact of bleaching agents on dental enamel microhardness. A Review. Dent. Mater..

[21] Borges B.C., Borges J.S., de Melo C.D., Pinheiro I.V., Santos A.J., Braz R., Montes M.A. (2011). Efficacy of a nivel at-home bleaching technique with carbamide peroxides modified by CPP-ACP and its effect on the microhardness of bleached enamel. Oper. Dent..

[22] de Vasconcelos A.A., Cunha A.G., Borges B.C., Vitoriano Jde O., Alves-Júnior C., Machado C.T., dos Santos A.J. (2012). Enamel properties after tooth bleaching with hydrogen carbamide peroxides in association with a CPP-ACP paste. Acta Odontolgoica Scand..

[23] Sanz-Sánchez I., Oteo-Calatayud J., Serrano J., Martín C., Herrera D. (2019). Changes in plaque and gingivitis levels after tooth bleaching: A systematic review. Int. J. Dent. Hyg..

[24] (2020). Review Manager (RevMan) [Computer Program].

[25] Page M.J., McKenzie J.E., Bossuyt P.M., Boutron I., Hoffmann T.C., Mulrow C.D., Shamseer L., Tetzlaff J.M., Akl E.A., Brennan S.E. (2021). The PRISMA 2020 statement: An updated guideline for reporting systematic reviews. BMJ.

[26] Sterne J.A.C., Savović J., Page M.J., Elbers R.G., Blencowe N.S., Boutron I., Cates C.J., Cheng H.Y., Corbett M.S., Eldridge S.M. (2019). RoB 2: A revised tool for assessing risk of bias in randomised trials. BMJ.

[27] Sterne J.A.C., Hernán M.A., Reeves B.C., Savović J., Berkman N.D., Viswanathan M., Henry D., Altman D.G., Ansari M.T., Boutron I. (2016). ROBINS-I: A tool for assessing risk of bias in non-randomized studies of interventions. BMJ.

[28] Guyatt G.H., Oxman A.D., Vist G.E., Kunz R., Falck-Ytter Y., Alonso-Coello P., Schünemann H.J., GRADE Working Group (2008). GRADE: An emerging consensus on rating quality of evidence and strength of recommendations. BMJ.

[29] Hosoya N., Honda K., Iino F., Arai T. (2003). Changes in enamel surface roughness and adhesion of Streptococcus mutans to enamel after vital bleaching. J. Dent..

[30] Zhang B., Huo S., Liu S., Zou L., Cheng L., Zhou X., Li M. (2021). Effects of Cold-Light Bleaching on Enamel Surface and Adhesion of Streptococcus mutans. Biomed. Res. Int..

[31] Hou X., Yuan K., Huang Z., Ma R. (2021). Effects of Bleaching Associated with Er:YAG and Nd:YAG Laser on Enamel Structure and Bacterial Biofilm Formation. Scanning.

[32] Voina C., Delean A., Muresan A., Valeanu M., Mazilu Moldovan A., Popescu V., Petean I., Ene R., Moldovan M., Pandrea S. (2020). Antimicrobial Activity and the Effect of Green Tea Experimental Gels on Teeth Surfaces. Coatings.

[33] Ittatirut S., Matangkasombut O., Thanyasrisung P. (2014). In-office bleaching gel with 35% hydrogen peroxide enhanced biofilm formation of early colonizing streptococci on human enamel. J. Dent..

[34] Al-Qunaian T.A. (2005). The effect of whitening agents on caries susceptibility of human enamel. Oper. Dent..

[35] Nam S.H., Ok S.M., Kim G.C. (2018). Tooth bleaching with low-temperature plasma lowers surface roughness and Streptococcus mutans adhesion. Int. Endod. J..

[36] Kim Y., Son H.H., Yi K., Ahn J.S., Chang J. (2016). Bleaching Effects on Color, Chemical, and Mechanical Properties of White Spot Lesions. Oper. Dent..

[37] Al-Angari S.S., Lippert F., Platt J.A., Eckert G.J., González-Cabezas C., Li Y., Hara A.T. (2019). Bleaching of simulated stained-remineralized caries lesions in vitro. Clin. Oral. Investig..

[38] Briso A., Silva Ú., Souza M., Rahal V., Jardim Júnior E.G., Cintra L. (2018). A clinical, randomized study on the influence of dental whitening on Streptococcus mutans population. Aust. Dent. J..

[39] Jansen E.E., Meyer-Lueckel H., Esteves-Oliveira M., Wierichs R.J. (2021). Do bleaching gels affect the stability of the masking and caries-arresting effects of caries infiltration-in vitro. Clin. Oral. Investig..

[40] Pinto C.F., Paes Leme A.F., Cavalli V., Giannini M. (2009). Effect of 10% carbamide peroxide bleaching on sound and artificial enamel carious lesions. Braz. Dent. J..

[41] Berger S.B., Pavan S., Dos Santos P.H., Giannini M., Bedran-Russo A.K. (2012). Effect of bleaching on sound enamel and with early artificial caries lesions using confocal laser microscopy. Braz. Dent. J..

[42] Ogura K., Tanaka R., Shibata Y., Miyazaki T., Hisamitsu H. (2013). In vitro demineralization of tooth enamel subjected to two whitening regimens. J. Am. Dent. Assoc..

[43] Lee J., Okoye L.O., Lima P.P., Gakunga P.T., Amaechi B.T. (2020). Investigation of the esthetic outcomes of white spot lesion treatments. Niger. J. Clin. Pract..

[44] Al-Angari S.S., Lippert F., Platt J.A., Eckert G.J., González-Cabezas C., Li Y., Hara A.T. (2019). Dental bleaching efficacy and impact on demineralization susceptibility of simulated stained-remineralized caries lesions. J. Dent..

[45] Alves E.A., Alves F.K., Campos Ede J., Mathias P. (2007). Susceptibility to carieslike lesions after dental bleaching with different techniques. Quintessence Int..

[46] Al-Shahrani A.A., Levon J.A., Hara A.T., Tang Q., Lippert F. (2020). The ability of dual whitening anti-caries mouthrinses to remove extrinsic staining and enhance caries lesion remineralization—An in vitro study. J. Dent..

[47] Pretty I.A., Edgar W.M., Higham S.M. (2005). The effect of bleaching on enamel susceptibility to acid erosion and demineralisation. Br. Dent. J..

[48] Mor C., Steinberg D., Dogan H., Rotstein I. (1998). Bacterial adherence to bleached surfaces of composite resin in vitro. Oral. Surg. Oral. Med. Oral. Pathol. Oral. Radiol. Endod..

[49] Steinberg D., Mor C., Dogan H., Zacks B., Rotstein I. (1999). Effect of salivary biofilm on the adherence of oral bacteria to bleached and non-bleached restorative material. Dent. Mater..

[50] Wongpraparatana I., Matangkasombut O., Thanyasrisung P., Panich M. (2018). Effect of Vital Tooth Bleaching on Surface Roughness and Streptococcal Biofilm Formation on Direct Tooth-Colored Restorative Materials. Oper. Dent..

[51] Gurgan S., Bolay S., Alaçam R. (1996). Antibacterial activity of 10% carbamide peroxide bleaching agents. J. Endod..

[52] Zheng C.Y., Pan J., Wang L., Zhang C.F. (2011). Effects of hydrogen peroxide-containing bleaching on cariogenic bacteria and plaque accumulation. Chin. J. Dent. Res..

[53] Briso A.L., Gonçalves R.S., Costa F.B., Gallinari M.d.e.O., Cintra L.T., Santos P.H. (2015). Demineralization and hydrogen peroxide penetration in teeth with incipient lesions. Braz. Dent. J..

[54] Alkmin Y.T., Sartorelli R., Flório F.M., Basting R.T. (2005). Comparative study of the effects of two bleaching agents on oral microbiota. Oper. Dent..

[55] Knösel M., Attin R., Becker K., Attin T. (2007). External bleaching effect on the color and luminosity of inactive white-spot lesions after fixed orthodontic appliances. Angle Orthod..

[56] Franz-Montan M., Ramacciato J.C., Rodrigues J.A., Marchi G.M., Rosalen P.L., Groppo F.C. (2009). The effect of combined bleaching techniques on oral microbiota. Indian J. Dent. Res..

